# Are you weary of illness? Coping and existential positioning in life with myasthenia gravis

**DOI:** 10.1007/s11019-026-10332-8

**Published:** 2026-02-23

**Authors:** Malene Missel, Nanna Witting, Malene Beck

**Affiliations:** 1 Department of Cardiothoracic surgery, Copenhagen University Hospital–Rigshospitalet, Blegdamsvej 9, Copenhagen, 2100 Denmark; 2 Department of Clinical Medicine , University of Copenhagen , Copenhagen, Denmark; 3 Copenhagen Neuromuscular Center, Department of Neurology, Rigshospitalet, Copenhagen University Hospital–Rigshospitalet, Copenhagen, Denmark; 4 Children and Adolescent Department, Research Unit, Zealand University Hospital, Roskilde, Denmark; 5 Faculty of Health, Institute of Regional Health, University of Southern Denmark, Odense, Denmark

**Keywords:** Myasthenia gravis, Chronic illness, Phenomenology, Autoethnography, Existential positioning, Illness weariness

## Abstract

This article explores what it means to position oneself existentially in relation to illness weariness in myasthenia gravis. Grounded in phenomenology and informed by autoethnographic principles, the study examines how the body’s changing disposition shapes lived meaning and coping in chronic illness. Phenomenological vignettes serve as experiential entry points, followed by reflective interpretation inspired by Havi Carel, Maurice Merleau-Ponty, Drew Leder, and Kay Toombs. Two experiential themes emerge. First, *When the body doesn’t always follow one’s lead anymore* shows how myasthenia gravis disrupts habitual rhythm and bodily trust. Bodily hesitation and the oscillation between resistance and responsiveness give rise to illness weariness-an existential modality distinct from physical fatigue or mood. Within this terrain, uncompromisingness appears as a quiet, existential stance; a refusal to let illness fully determine the direction of one’s life, expressed through a persistent insistence on participation and dignity. Second, *Physical activity and relational support as existential coping practices* demonstrates how movement and relationship sustain existential positioning. Physical activity becomes an existential practice through which hope, orientation, and embodied rhythm are maintained, not by erasing illness weariness but by placing it alongside an uncompromising insistence on participation. At the same time, relational support, here exemplified by an attuned neurologist, serves as a subtle relational nudge that grounds and strengthens the will to continue. The study conceptualizes illness weariness and uncompromisingness as a continuum of existential responses shaped by bodily fluctuation and relational context. These insights extend beyond myasthenia gravis and point to the importance of existential attentiveness and relational continuity in clinical care.

*Are you weary of illness?* she asked. An attuned and attentive neurologist. Her voice carried no judgement, only care. The question struck something I hadn’t seen myself. Was I tired *of* the illness? Of the fight, the medication, the system – or tired *within* it? Worn down, shaped by it, as if it had settled into the core of who I was? She put words to something I hadn’t been able to articulate. Something that had long been present, but never spoken. Her question offered no answers. It opened a space. *Are you weary of illness?* The words stayed with me. Quietly working. A gentle but persistent challenge; How do you live with illness as a condition of life, without losing yourself? How do you hold on to movement, direction, and dignity when your body won’t always follow? Over the past four years – with a body that sometimes holds back, and in the ongoing effort to maintain an active life – the quiet yet unwavering encouragement from this neurologist have sparked reflections. This text is not an answer, but a movement toward understanding what it means to live with the chronic illness myasthenia gravis as an existential challenge.

## Introduction

*Pain is inevitable. Suffering is optional.* Haruki Murakami (2009) writes this in his book *What I Talk About When I Talk About Running*. The book revolves around willpower, endurance, and movement – and about holding on to oneself in the world, one step at a time. Perhaps it speaks to something that otherwise resists words. Taking Murakami’s words as a quiet prompt, this article extends the reflection by venturing into the terrain of chronic illness – where endurance is not measured in distance, but in the ongoing effort to hold on to meaning, identity, and direction.

The focus is an exploration of the phenomenon referred to in Danish as *sygdomstræthed* – literally, *illness tiredness*, but more accurately captured by the term *illness weariness*. Interestingly, *sygdomstræthed* is not a formal medical term in Danish, but a word coined by a neurologist in a clinical encounter – a term that, while informal, has since proven resonant across both clinical and experiential contexts. It captured something otherwise difficult to name – a sense of being worn down not only by symptoms, but by the ongoing demands of living with illness as an existential condition. This exploration is about insisting – on movement, on meaning, on oneself – in the midst of daily life that has to carry on, even when the body holds back. What is at stake is not tiredness as a symptom, but existential positioning as a lived practice in a life shaped by myasthenia gravis.

## Approaching the phenomenon

Life with chronic illness is not merely a medical condition, but also an existential experience (Beck et al. 2023; Charmaz 1997; Laranjeira and Dourado 2022; Larsen et al. 2018; Missel et al. 2021). For many, it is not only a matter of symptoms and treatment, but of the quiet, ongoing negotiations of daily life with a body that no longer responds as it once did. A subtle kind of resistance may arise – not dramatic, but enduring – shaping one’s way of being in the world (Aho 2018; Carel, 2017; Charmaz 2000; Frank 1995; Toombs 2001). The healthcare system often operates within classical paradigms that frame illness as a deviation to be corrected, with treatment focused on measurable symptoms and levels of functioning (Chochinov 2022; Duffee 2023; Frank 2019; Gardner and Kleinman 2019). Yet the lived experience carries something more – something that does not easily lend itself to measurement or testing.

Fatigue is a recurring theme in many illness trajectories and is often understood in health sciences as a complex and multidimensional phenomenon – encompassing physical, cognitive, and emotional exhaustion that may arise as a consequence of illness, treatment, or both (Knoop et al. 2023; Machado et al. 2021; Twomey et al. 2017). In research and clinical practice, a distinction is often made between subjective fatigue and fatigability; the self-reported perception of fatigue and the objectively measurable reduction in performance over time (Kluger et al. 2013; Penner and Paul 2017). In conditions such as myasthenia gravis (Salari et al. 2021; Vissing et al., 2024) fatigue is a central yet difficult-to-define symptom (Andersen et al. 2021, 2022; Berrih-Aknin et al. 2023; Dewilde et al. 2024). It involves both muscle fatigability that worsens with activity, and a broader experience of mental fatigue that does not necessarily improve with rest (Akkan et al., 2022). Myasthenia gravis is a chronic autoimmune neuromuscular disorder characterized by varying degrees of skeletal muscle weakness caused by a breakdown in the normal communication between nerves and muscles (Berrih-Aknin et al. 2023; Gilhus et al., 2024). The symptoms arise when autoantibodies bind to and disrupt postsynaptic receptors, thereby reducing the efficiency of synaptic signaling. This leads to fluctuating muscle weakness that typically worsens with activity and improves with rest. All skeletal muscle groups may be affected, resulting in symptoms such as ptosis, diplopia, dysarthria, difficulty chewing and swallowing, shortness of breath, and weakness in the extremities.

In healthcare, the severity of illness is often assessed based on whether it is life-threatening, treatable, or objectively measurable. Myasthenia gravis is an objectively measurable disease and is often perceived as a relatively *manageable* condition, where symptoms can be kept under control through medical treatment (Farmakidis et al. 2018; Narayanaswami et al. 2021). But the clinical picture does not tell the whole story. Even when the disease appears *controlled* from the outside, the lived experience may be marked by uncertainty, vulnerability, exhaustion, and the ongoing work of renegotiating one’s relationship with the body and with oneself (Hartford et al. 2023; Jackson et al. 2023; Keer-Keer 2015; Olesen et al. 2025). As Havi Carel (2017) notes, such experiences often arise when bodily certainty is disrupted and gives way to *bodily doubt*; a shift that reshapes how one moves, acts, and understands oneself in daily life. Carel (2024), in her article *Breaking the Silence on Illness*, also draws attention to the lack of language and space for those dimensions of illness that cannot be measured but are *felt*. Illness can give rise to a kind of silence, both in the patient’s own approach, and in encounters with the healthcare system, where the existential and embodied dimensions may be overlooked (Ahlzén 2011; Kidd & Carel, 2017; Svenaeus 2017). By exploring and articulating these experiences, a voice is given to what often remains unspoken, yet shapes life with illness (Frank 2020). This article thus seeks to shed light on how even a *treatable* chronic illness such as myasthenia gravis can be existentially intrusive.

In healthcare practice, coping is often understood within psychological frameworks, such as the stress and coping model by Richard S. Lazarus and Susan Folkman (1988; Folkman 1984, 2008), Aaron Antonowsky’s (1982; Vinje et al. 2022) concept on sense of coherence, or Howard Leventhal’s (Hagger and Orbell 2022; Leventhal et al. 2016) model of illness representations. While these approaches have shaped clinical practice, they primarily describe cognitive and functional strategies aimed at adaptation, problem-solving, or emotional regulation. However, they offer limited language for the existential, embodied and everyday dimensions that shape life with chronic illness. In this article, *uncompromisingness* is introduced as an existential form of coping – not denial, but a persistent insistence on participation, integrity and dignity, even when illness challenges one’s sense of self and capacity to act.

This article explores what it means to position oneself existentially in relation to illness. How does one hold on to oneself when the body resists the intentions? And how can physical activity – as in this lived experience – become a way of responding to that struggle? Not to defeat the illness, but to insist on life. Although grounded in personal experiences with myasthenia gravis, the questions raised extend beyond this particular condition and speak to the broader phenomenon of illness weariness. The focus is on lived experience and on forms of coping that may at times align with healthcare understanding – yet also move beyond established clinical frameworks.

## Methodology

This study is grounded in an applied phenomenological approach (Fernandez 2020; Gallagher & Sørensen, 2006; Zahavi and Martiny 2019), aiming to explore the lived, embodied, and existential dimensions of chronic illness in everyday life. The article integrates autoethnographic elements in the sense that the first authors lived experience with myasthenia gravis forms the experiential point of departure for the phenomenological inquiry. In this context, the narrative serves as a situated entry point into the phenomenon of illness weariness. This combination of phenomenology and first-person reflection enables an open, embodied, and contextually anchored exploration of how illness shapes meaning, existential coping, and being-in-the-world. The following section outlines the philosophical and methodological principles guiding this approach.

### Philosophical and methodological foundations

#### Phenomenological approach: lifeworld, embodiment, and existential meaning

This study is philosophically grounded in a phenomenological understanding of the lifeworld, embodied experience, and the existential meaning that emerges through illness (Gallagher and Zahavi 2020; Merleau-Ponty 2002, 2004; Zahavi 2018; Zahavi and Martiny 2019). Through a phenomenological lens, the focus is on how illness is experienced *from within* – as a disruption of the taken-for-granted nature of existence and the familiarity of the body. At the heart of this approach is the notion of the body as an anchor in the world (Merleau-Ponty 2002), understood as the primary ground for experience and the emergence of meaning. Perception is not passive reception but a bodily attunement to significance, shaped by emotions, intentions, and social context. Even ambiguous, resistant, or disruptive bodily states are therefore meaningful; they reveal how illness alters one’s relation to self, world, and others (Merleau-Ponty 2002, 2004).

Building on this foundation, Max van Manen’s (2016) view of *phenomenological writing* as a pathway for cultivating insight brings into view the role of attentive description in revealing the depth of lived experience. In line with Kevin A. Aho’s (2018) notion of existential medicine, phenomenology here becomes a way of uncovering the existential dimensions of illness and the meaning-bearing nature of illness weariness. This perspective enables an exploration of illness weariness and uncompromisingness as lifeworld phenomena that unfold in the dynamic interplay between body, relationships, and existential positioning. Knud E. Løgstrup’s (1991) reflections on the sensuous and ethical dimensions of human existence offer a further resonance space, highlighting how vulnerability, attentiveness, and the ethical-existential presence of the other shape the lived experience of illness.

#### Autoethnographic elements: experience as a source of knowledge

Alongside its phenomenological foundations, this study draws on autoethnographic principles in which the researcher’s own experiences with myasthenia gravis serve as a legitimate source of insight and analytical depth. The aim is not autobiographical storytelling for its own sake, but to use this situated, first-person experience to illuminate a broader existential phenomenon; what it means to live with a chronic and fluctuating illness.

This approach aligns with analytical autoethnography (Bochner and Ellis 2016; Ellis et al. 2011; Wall 2006), where the researcher is both an embodied participant and a reflective analyst. It involves a methodological and ethical commitment to reflexivity and to making the researcher’s position explicit. As Donna Haraway (1988, 2022) reminds us, knowledge is always situated; there is no neutral vantage point from which illness can be known. Integrating phenomenological insights on embodied first-person experience with autoethnographic reflexivity creates a hybrid space of inquiry in which lived experience becomes an epistemic resource rather than a limitation. This position fosters epistemic humility and acknowledges that vulnerability and bodily knowledge can expand conventional understandings of illness (Carel, 2017; Kidd and Carel 2025).

The first-person experience is conveyed in an open linguistic form – not through *I* but through a narrative voice intended to make the experience accessible to the reader. This stylistic choice and aesthetic move maintain a balance between proximity and analytical distance. As Carel (2011, 2014, 2017) notes, such writing can reveal what is *universally possible in human experience*; not universal in a statistical or normative sense, but as an existential structure that can resonate beyond the individual case. Through this transformation, a singular account becomes a reflective mode of access to experiences that others may recognize or find meaningful. In this way, the text remains rooted in phenomenological first-person experience (Fernandez 2020; Zahavi and Martiny 2019) while creating space for individual vulnerability, embodiment, and reflection to contribute to a broader understanding of illness weariness as an existential phenomenon.

### Researcher positioning

#### Situated experience and epistemic humility

Living with myasthenia gravis while also working as a nurse and researcher creates a particular form of situated knowledge (Haraway 1988, 2022). The body is not only an object of clinical understanding but the lived point of departure for reflection. This dual position means that vulnerability, uncertainty, illness weariness, and moments of uncompromisingness are not merely observed but *felt*, and the alternation between experiential insight and analytical interpretation becomes a natural mode of inquiry. As Carel (2014) reflects, the lived experience of illness differs fundamentally from third-person descriptions; it alters the body, the self, and one’s orientation in the world simultaneously.

From this position, understanding emerges not in isolation but through the interplay between embodied experience, conceptual reflection, and relational encounters. Exchanges with the neurologist – marked by clarity, honesty, and steady presence – have shaped both the lived experience of myasthenia gravis and the analytical movement of this article. Such encounters can open a reflective space in which one is not only treated but invited to think, feel, and take a stand (Frank 1995; Løgstrup 1991). Working from this standpoint requires epistemic humility; an awareness that knowledge is always partial, embodied, and situated, and that lived experience can challenge and expand established assumptions (Carel, 2017, 2021; Kidd and Carel 2025). Within this study, the dual perspective – being both the sensing body and the reflecting researcher – forms a legitimate epistemic position from which to explore illness weariness, uncompromisingness as a form of existential coping, and the dynamics of existential positioning.

### Theoretical inspiration

To capture and illuminate the embodied and existential aspects of living with myasthenia gravis, the analysis draws on phenomenological theory. Instead of functioning as an abstract framework, these perspectives provide a way of seeing and articulating lived experience – offering concepts and sensitivities that make it possible to describe the tensions, ambiguities, and meaning structures that accompany life with myasthenia gravis.

Carel and Ian James Kidd's (2017, 2021, 2025) work on illness as a way of being-in-the-world, including concepts such as bodily doubt and epistemic injustice, offers a lens for understanding the uncertainty that arises when bodily reliability is disrupted. Maurice Merleau-Ponty’s (1973, 2002, 2004) account of embodiment as our existential grounding informs how illness affects perception, existential coping, and orientation. Drew Leder’s (1990) descriptions of *dis*-appearance and *dys*-appearance capture how the body enters awareness through resistance, while his later work on *re*-attunement – sometimes described as a form of bodily *re*-appearance – highlights the possibility of renewed bodily connection even within suffering (Leder 2023). Kay Toombs (1992, 2001, 2008) further contributes an experiential understanding of illness as existential disruption, emphasizing how the once taken-for-granted body becomes unfamiliar and how this reconfigures identity and everyday life.

Together, these perspectives support the conceptualization of illness weariness and uncompromisingness as embodied and existential phenomena that emerge in the dynamic interplay between the body, the self, and the world.

## Vignette-based approach

### Voice, form, and interpretive movement

The applied combination of phenomenological insight and autoethnographic elements shapes both the text and the reflective interpretation (Bochner and Ellis 2016; Carel 2021; Finlay 2011; van Manen 2016). The interpretive movement unfolds through two intertwined modes; *phenomenological vignettes*, which offer situated descriptions of lived moments, and *reflective interpretations* which open these experiences toward existential understanding. The vignettes are not presented as personal anecdotes or clinical cases; rather, they serve as concentrated, felt glimpses – experiential windows that allow the reader to sense the phenomenological texture of the bodily and existential movements that may characterize life with myasthenia gravis. The reflective sections following each set of vignettes bring these lived moments into dialogue with phenomenological perspectives. Rather than applying theory from the outside, the reflections articulate meanings that arise from within the experience itself. The relationship with the neurologist – both clinically and intellectually attuned – is acknowledged as part of the contextual ground that shapes understanding. Rather than data, this relationship serves as a condition that enables reflection and resonance (Frank 1995, 2012).

Taken together, the vignettes and reflective interpretations constitute a mode of inquiry in which embodied experience, linguistic articulation, and philosophical interpretation remain closely connected. This form allows the study to explore illness weariness, uncompromisingness as an existential form of coping, and the dynamics of positioning oneself in relation to a fluctuating body – not as abstract concepts, but as lived phenomena unfolding in the interplay between body, self, world, and relation. When experience and theory are brought into dialogue in this way, individual encounters with illness can reveal broader patterns and understandings that may resonate with others living with myasthenia gravis or other chronic illness. In the sections that follow, this structure becomes visible; each thematic part begins with a brief orientation, followed by phenomenological vignettes and reflective interpretation. This organization allows lived moments to unfold into existential insight and brings illness weariness, uncompromisingness, and the work of positioning oneself into clearer view.

### When the body doesn’t always follow one’s lead anymore

It is not an ordinary tiredness. It is the body hesitating – no longer following the familiar rhythm of exertion and recovery. What once felt like natural restoration becomes unpredictable. The body no longer returns to itself with ease. A form of bodily resistance settles in as an underlying tone in everyday life.

A sense arises that one can no longer *repair* oneself as before. Activity no longer necessarily makes one stronger, and rest sometimes feels like waiting for something that never arrives. The body has lost its immediate responsiveness and now reacts slowly, unevenly, reluctantly.

This is not about laziness, weakness, or resignation. On the contrary, there is often a continuous effort in trying – to mobilize will, structure, and clarity – only to be met with resistance. Not always overwhelming, but always present. A weight stitched into the days, not heavy enough to stop you, but enough to be felt. Not necessarily exhausted, but weighed down – by the body, by repetition, by the sense that possibilities have narrowed.

Perhaps this is illness weariness – not a mental fatigue marked by lack of clarity, but a diffuse exhaustion that seeps into the rhythm of everyday life. And still, one gets up, walks, tries again. Not because it is easy, but because one has not given up. Not yet. This persistence can be understood as a form of uncompromisingness – an existential defiance that refuses to let illness define the direction of one’s life. It is not a denial of limitations, but a continuous insistence on participation, on remaining oneself – even when the body pulls in another direction.

The existential experience of a body that no longer always follow one’s lead is explored in the following three phenomenological vignettes, each examining bodily sluggishness, an unexpected sense of flow, and the doubt and hesitation that arise when the body no longer feels transparent.

#### A body that doesn’t keep up

A morning like any other, yet the body feels strangely different. Not exactly ill, not entirely failing – just weighed down. As if the muscles are filled with something invisible. Usual movement begins, but the steps lack rhythm. Not because the legs don’t work, but because the connection between intention and movement falters. Something once felt given, the pace, the direction, the very effortlessness of movement, now seems to require renegotiation. An unexpected longing arises – not to perform, but simply for the body to join in. Just to be part of it. Now it feels like a companion slightly out of step – unpredictable, sluggish, perhaps even slightly offended. And still, the path is followed. Not out of joy, not out of ease – but because that is what is done. A fragile kind of insistence. One step at a time – without guarantees, only a quiet thread of hope.

#### When the body surprises

Standing in workout clothes, already sensing the weight of what’s to come. Lately, legs have failed, and the body has felt like a stranger. But this time, something shifts. The movements fall into place. The muscles respond – almost willingly. There is still resistance, but it is manageable. Something that can be moved with. Trust does not come easily. There is an expectation that the body will withdraw. But it doesn’t. It holds. The running strides become rhythmic – not light, but not heavy either. Air touches the skin, light gathers around, the body in motion – as an opening in the midst of what is often unpredictable. A sense of connection returns. It is not triumph. It is something else – a kind of gratitude. A temporary kinship between will and body, a step in rhythm with yourself.

#### When the body doesn’t yield

A comment from the neurologist lingers. *You’re not fully yielding*. Not as a criticism, but as an observation. And something about it feels true. For how does letting go even happen when the body can no longer be trusted? When running, exercising, swallowing, or speaking is accompanied by a constant internal check; is the body responding as it should? Awareness intensifies, perhaps too much. And in this gaze – which may resemble control but stems from doubt – a kind of resistance emerges. Not deliberate, not intentional, but because the body and the will are no longer in sync. Something that was once silent now demands attention. Standing in the tension between what is felt and what cannot easily be shown. Not out of unwillingness to yield, but out of no longer quite knowing how.

#### Reflections on bodily hesitation and illness weariness

In this lived terrain, the question *Are you weary of illness?* becomes more than a clinical inquiry – it becomes an existential invitation; an opening toward the existential dimensions of illness, inviting reflection on how life is lived with a vulnerable and unpredictable body. It points to a weariness that does not reside solely in the muscles, but seeps into the rhythm of life, casting doubt on one’s ability to keep going.

The vignettes point to a central aspect of life with myasthenia gravis; the body’s changing disposition. The experience does not unfold as a linear narrative of decline or improvement, but as a constant oscillation between resistance and responsiveness – between moments when the body holds back and moments when it once again cooperates. This ambiguity is not merely a physical condition, but an existential experience that requires ongoing interpretation, positioning, and meaning making. Movement becomes an existential gamble – an act made without any guarantee of how the body will answer.

From a phenomenological perspective, this can be understood as a disturbance in our ordinary being-in-the-world. For Merleau-Ponty (2002, 2004), the body is our primary way of being present in the world; it is through the body that we perceive, act, and find our orientation. When myasthenia gravis disrupts this tacit bodily familiarity, each step, each effort becomes charged with uncertainty. Carel (2011, 2017) describes this shift as bodily doubt – a loss of trust in the body’s reliability. What was previously taken for granted now requires continuous monitoring and negotiation. In the first vignette, this doubt appears as a hesitation between intention and movement; in the second, as a cautious gratitude when the body unexpectedly responds.

Toombs (1992, 2008) emphasizes how illness not only affects physical function but also transforms one’s mode of being in the world. The body, once a silent background, emerges into awareness as a resistant presence. Leder’s (1990) concept of *dys*-appearance captures this movement well; the body becomes noticeable precisely when it disrupts. With myasthenia gravis, *dys*-appearance is not a rare event but a recurrent condition – a repeated interruption of bodily transparency. Yet, as Leder (2023) later suggests, this bodily emergence is not only a source of suffering; it can also open a space for reflection and renewed contact with oneself. In this light, illness weariness can be understood as an existential modality arising from the continuous demand to negotiate with a body that is both with and against oneself. It is not simply physical exhaustion, but a subtle erosion of the ongoing work of meaning making. The weariness settles in the relation between body, self, and world – in the repeated experience of having to adjust, reinterpret, and reintegrate one’s possibilities. This is not necessarily hopelessness, but it is heavy.

At the same time, the vignettes show how a particular mode of being emerges in response to this condition. One gets up, runs, walks, tries again – not with a promise of improvement, but because giving up would mean losing an important part of oneself. Toombs (2008) describes this as an existential reconstruction; values, responsibilities, and self-understanding must be reworked in light of a changed body. Here, perseverance becomes more than a psychological trait; it can be seen as an existential strength. Svend Brinkmann’s (2014) notion of standing firm in uncertainty captures this stance – not as a heroic posture, but as a quiet willingness to remain in what is given. It is in this context that the concept of uncompromisingness becomes relevant. Uncompromisingness, as it appears in these experiences, is not denial of illness, but an insistence on participation and dignity. It is a refusal to let myasthenia gravis dictate the terms of one’s life, even while acknowledging its presence. Søren Kierkegaard’s (1962) idea of choosing oneself within the given resonates here; existential positioning is not about changing the conditions, but about how one chooses to live with them. In this sense, uncompromisingness can be seen as a form of existential coping – a way of maintaining identity, direction, and engagement amidst moments of bodily doubt.

Ambiguity plays a crucial role in this process. The body is neither simply an enemy nor a reliable friend, but both. It both enables and obstructs, appears and recedes. Merleau-Ponty (2002, 2004) underscores ambiguity as a fundamental condition of embodiment; in myasthenia gravis this ambiguity becomes particularly pronounced. The third vignette captures this tension in the neurologist’s comment about *not fully yielding*. It points to the difficulty of letting go when bodily trust has been repeatedly undermined. The apparent resistance is not a lack of will, but an expression of existential caution. In the interplay between illness weariness and uncompromisingness, an existential continuum becomes visible. Illness weariness marks the weight of ongoing bodily negotiation; uncompromisingness marks the active stance of remaining in life despite this weight. Both are responses to the same condition of bodily unpredictability. To live with myasthenia gravis is to live in this tension – and to continually find ways of positioning oneself within it.

### Physical activity and relational support as existential coping practices

As Carel (2011, 2012, 2017) argues, movement is not merely physical displacement but an existential act – a way of projecting oneself into the world. This form of existential movement becomes particularly significant in the context of myasthenia gravis, where the body no longer always functions effortlessly, and movement is often experienced as an act of will. Walking, running, or exercising thus becomes more than training; it becomes a way of remaining engaged in everyday life, of sustaining a connection between body and world.

Physical activity, in this sense, becomes a kind of practice philosophy – a daily negotiation with the body and a tacit response to its resistance. Not in order to control it, but to meet it where it is. Running, even when it barely feels like running, becomes an existential act – not in spite of myasthenia gravis, but with it. It is not about conquering the body, but about choosing to dwell in it, even when things are neither easy nor straightforward.

In this experiential terrain, physical activity can be understood as a response to myasthenia gravis’ uninvited pause. It becomes a way of holding on to participation when the body hesitates. And yet, the question lingers; *Are you weary of illness?* Asked with sincerity rather than suspicion, it pointed to a quiet unravelling that rests not only in the muscles but seeps into the rhythm of life – a form of weariness that challenges one’s ability to continue.

In this context, uncompromisingness is not a denial of vulnerability, but an expression of integrity – a will to insist on unfolding life, even when it entails adversity. There is an existential dignity in choosing to act, even when the outcome is uncertain, and the body yields only partially. This can be understood as a response to illness weariness – a way of standing up to an exhaustion that is not merely physical but existential. Within this space, the encounter with a clinician whose presence is attuned and trustworthy becomes significant. Through gentle nudges and steady continuity, it becomes possible to shift; not away from illness, but toward a more honest acknowledgment of life with myasthenia gravis.

The existential significance of movement – as both embodied expression and relational support – is explored in the following three vignettes.

#### Legs that tire slowly – yet with dignity

A run begins, fully aware that the body may choose to protest from the very first step. This is not ordinary fatigue, but a slow and persistent resistance that settles deep in the thighs. The muscles feel as though they are weighted down by something invisible, and each step grows increasingly resistant, until the movement halts on its own; when the legs simply refuse to move further. A delay arises between will and reality, as though the body must be convinced with every step. And yet the movement continues. Not in the hope that it will become easier, but because something within insists. There is a kind of dignity in continuing; a slow but proud response to the body’s hesitation. In this very slowness, a space for contemplation opens – not as reflection detached from the body, but as a felt sense of what it means to remain in life, even when it costs. This is not tiredness, not just fatigability, but an existential anchoring in what is.

#### Gentle nudges and mutual trust

In the encounter with a clinician who knows the patient over time, a particular kind of trust can emerge. Not trust as blind reassurance, but as something active and mutual. When the neurologist not only asks about symptoms, but invites reflection and sensing, a space opens in which it is possible to be both patient and person. At times, something is articulated that has long been felt but lacked words. At other times, questions are asked not to be answered, but to move something within. When the body or self-image resists change, this encounter becomes a place where that resistance can be held – and gently challenged. It is not a direct intervention, but a gentle nudge, a kind of respectful guidance that makes it possible to stay true to oneself, even in vulnerability. Openings and shifts occur not because of pressure, but because recognition is offered. Such an encounter makes it possible to contain the complexity of living with illness – and still feel whole.

#### Held by clarity and direction

There are many questions. Not because worry dominates, but because understanding matters. Understanding the illness – physiologically and practically. Understand the connections between symptoms. Understand what the body is doing, and why. Understand – in order to endure it. Messages are written to the neurologist. Many messages. Sometimes several in a row. And the reply comes – always. Brief. Precise. With a clarity that creates a sense of safety. Not warmth in the conventional sense, but a clear care that shines through. It is not just information – it is direction. In the consultation, the tone is the same; sober, focused, yet never rushed. There is a calm presence, with attention to what matters, to the whole, and to the person behind the symptoms. And that makes a difference. It creates space for both doubt and trust. Words do not always need to be found. They are already there – in the answers, and in the quiet reassurance that arises when feeling seen, even without having to explain everything.

#### Reflections on movement, relational nudge, and illness weariness

In the daily movement – in the repetition of steps, the rhythm of breathing, the familiar path – a sense of recognizability emerges that can carry the work of meaning. It is not about performance, but about staying in motion. When the body responds, even slightly, an experience of connection returns; of still being in the world. Murakami’s (2009) reflections on running as an existential practice resonate here; one does not run to win, but to sustain a rhythm, a direction, a sense of self. In this returning to movement, with myasthenia gravis as a companion, a quiet form of hope becomes possible – not as a promise of cure, but as the lived sense that much remains open. In this light, physical activity can be understood as an existential response to illness weariness – not by erasing it, but by placing it alongside an uncompromising insistence on participation.

From a phenomenological perspective, repetition and rhythm become a form of existential anchoring in a life marked by vulnerability and a body out of rhythm. For a person living with myasthenia gravis, repetition is not merely a physical pattern, but a bodily gesture imbued with meaning – a step, a breath, a rhythm upheld in the face of resistance. Here, Carel’s work on repair (2017), Kidd and Carel (2021) provides a useful lens; repair is not cure, but the ongoing work of re-establishing orientation in the world. Toombs (1992, 2008) similarly underscores how routines that once were automatic become points of orientation that help sustain coherence and belonging. Rhythmic movement, seen this way, becomes an existential answer to illness weariness; not a sign of surplus energy, but of will. The daily insistence on movement expresses a form of existential integrity; a persistence that affirms that even when the body fails, there remains something one actively chooses to stay connected to. Leder (2023) describes such gestures as acts of *re*-attunement – not naive attempts to *return to normal*, but embodied ways of gradually restoring trust.

When the body, despite reluctance, still responds, a momentary sense of presence arises. Leder (1990) describes this as a movement from *dys*-appearance toward what he later terms *re*-appearance (Leder 2023) – where the body, once experienced mainly as problematic, again becomes a collaborator. These moments may be brief, but they reaffirm the connection between body, self and world. Leder (2023) emphasizes how such moments can function as bodily points of contact where attention moves from control to relation. With myasthenia gravis, the body is no longer simply something to be managed or brought back to normal. It becomes a place where subjectivity and world reconnect through presence and openness. Toombs (1992, 2008) similarly shows how short instants of bodily attunement – when the body once again feels like a home, even if only for a while – can nurture hope and counteract alienation. Both Toombs and Carel understand these moments as existential repair; not the end of struggle, but the reappearance of something meaningful within it. Hope, in this context, does not take the form of future expectations but emerges as a bodily felt possibility in the present – a sense that everyday life can still unfold. Carel (2012, 2017) describes hope as something that can arise in action itself, as a way of standing in uncertainty while remaining engaged in life. Irvin D. Yalom (1980) likewise notes that hope is often generated relationally and through action. In this sense, movement – running, walking, rhythm – functions as an existential practice through which connection to self and others is sustained. Uncompromisingness can here be understood as a gesture of hope; a persistent insistence on participation, even when the body hesitates. Hope is not merely thought but lived.

The relationship with the neurologist becomes, in this perspective, a form of existential grounding. In the encounter with a knowledgeable and relationally attentive other, a space emerges in which doubt, questions, and bodily uncertainty can be held without always needing to be resolved. Brief replies, steady presence, and clinical clarity are not merely informative, but orienting. Løgstrup’s (1991, 1996) reflections of spontaneous and sovereign expressions of life – such as trust and openness – offers a way to understand what unfolds in such encounters. The neurologist’s ongoing presence can be seen as a quiet, steadfast presence rather than sentimentality; a way of *carrying something of the patient’s life in her hands* (Løgstrup 1991). Brinkmann’s (2014) notion of steadfastness helps conceptualize how such relational continuity supports the ability to stand firm in uncertainty. Toombs (1987, 1992) shows how being met as a *whole person* within healthcare can sustain dignity and agency. Emmanuel Levinas (1969) reminds us that the encounter with the Other calls one to responsibility and presence. In this light, relational support is not merely an add-on, but part of what makes subjectivity possible; one’s sense of self takes shape in and through the way one is met by the Other. Seen this way, both illness weariness and uncompromisingness emerge not only from bodily conditions but also from relational contexts. When energy is worn thin and illness weariness threatens to undermine initiative, relational support can function as an existential push. It does not necessarily provide new energy but anchors the will to continue. Uncompromisingness is therefore not a solitary act, but a supported insistence. Movement is guided not only by the body but also by how one is met.

In this way, physical activity and human connection together form a meaning-bearing practice in life with myasthenia gravis – a quiet yet persistent response to the body’s changing disposition. Illness weariness and uncompromisingness can thus be understood as existential responses along the same continuum; illness weariness as the experience of the fading of existential coherence, and uncompromisingness as an active form of existential positioning. Within this continuum, movement and relational support become carriers of existential meaning, making possible an insistence on life even in conditions that are clinically manageable.

## From experiential insight to conceptual contribution and clinical relevance

The straightforward answer to the neurologist’s question, *Are you weary of illness?*, is simply *yes*. Living with even a *treatable* chronic condition like myasthenia gravis involves not only bodily vulnerabilities, but also existential negotiations. Yet, the weariness explored in this article is not simply equivalent to fatigue, low mood, or lack of motivation (Grahek et al. 2019; Kluger et al. 2013). Illness weariness, as developed here, refers to a phenomenological and existential modality; a slow depletion that settles between body, self, and world when myasthenia gravis continually interrupts bodily trust, rhythms of daily life, and the felt sense of continuity (Aho 2018; Carel, 2017; Merleau-Ponty 2002). It differs from physical fatigability, cognitive exhaustion, or depressive tiredness by being rooted in the ongoing work of meaning-making required by a fluctuating body. Alongside this, the study introduces uncompromisingness as a form of existential coping. This term is chosen deliberately; unlike persistence or determination, uncompromisingness captures a stance rather than a personality trait – a mode of insisting on participation, integrity and dignity even when the body hesitates (Brinkmann 2014; Kierkegaard 1962). It is not a normative ideal and is not available to everyone at all times. Rather, uncompromisingness emerges as a situational, relational, and bodily anchored response that coexists with vulnerability. Importantly, it is not the opposite of illness weariness; the two form a continuum. *Illness weariness* reflects the erosion produced by a changing bodily rhythm, while *uncompromisingness* shows how it might be possible to actively position oneself within that erosion. Both arise from the same existential ground.

The findings also make visible that uncompromisingness is not the only existential response available in life with myasthenia gravis or other chronic conditions. For some, coping may emerge through relational connection, faith, aesthetics, silence, nature, or practices of care (Beck et al. 2023; Koopman et al. 2016; Ladores et al. 2022; Missel et al. 2021). What matters is not the activity itself, but the meaning it holds as a way of stepping forward in life with illness. The analysis equally highlights that such existential coping is not a solitary achievement. Relational support, here exemplified by the ongoing, precise, and attuned responses with the neurologist, can function as an existential nudge. Not by offering reassurance or solutions, but by offering presence, clarity, and recognition. Without such relational grounding, the capacity for uncompromisingness may be weakened, and illness weariness may intensify (Levinas 1969; Løgstrup 1991). Obstacles such as fragmented care, lack of relational continuity, or overly clinical communication may therefore hinder existential coping and deepen weariness (Kidd and Carel 2025; Kidd & Carel, 2017).

Situated within phenomenological scholarship, this article contributes conceptual clarity by placing illness weariness in dialogue with, yet distinct from, existing concepts such as Toombs’ (1992) notion of *existential fatigue*. Toombs describes existential fatigue as a pervasive weariness arising from the loss of the body’s taken-for-granted transparency and the ongoing burden of being-in-the-world with illness. While this resonates with the experiences described in the present article, *illness weariness* differs in that it does not primarily stem from existential suffering alone, but from the continuous effort to live, participate, and maintain meaning in everyday life with a fluctuating and unreliable body. In this sense, illness weariness reflects not only vulnerability, but also the erosion produced by sustained existential work – that is, by the ongoing effort to live forward with illness, an effort shaped not only by bodily conditions but also by how one is met and supported within relational contexts. The article further places this concept in dialogue with Leder’s (1990) analyses of *dys*-appearance and later concept of *re*-attunement (Leder 2023), and Carel’s (2017) descriptions of *bodily doubt*. The notion of *uncompromisingness* extends the field by articulating an existential stance not adequately captured by existing terms of resilience, adaptation, or compliance. Through these two concepts, the article advances phenomenological understandings of how people actively orient themselves amid the body’s changing disposition – an orientation that combines weariness with the will to remain in life.

Although developed through the lens of myasthenia gravis, the concepts of illness weariness and uncompromisingness reach beyond this particular condition. Many chronic and fluctuating illnesses – long-term cancer survivorship (Frank and Solbraekke 2023), autoimmune disease (Herbrechter and Jamieson 2020), neurological disorders (Toombs 2001), and other forms of embodied vulnerability – may involve similar movements between *illness weariness* and *uncompromisingness*, between depletion and existential positioning. These concepts may therefore help illuminate lived experiences across diagnostic boundaries.

Clinically, these insights point to the importance of recognizing existential dimensions not as additions to care but as inherent to the lived experience of chronic illness. Physical activity, relational continuity, or persistent daily practices should not be dismissed as denial or unrealistic striving but understood as ways of maintaining meaning and anchoring oneself in life (Aho 2018; Carel 2014). Healthcare professionals are therefore invited to adopt a stance of epistemic humility; acknowledging that experiential knowledge is not bias but a legitimate form of understanding (Haraway 2022; Kidd & Carel, 2017). This calls for attentiveness to what may not be captured in clinical tests or symptom-oriented conversations.

Finally, the question *Are you weary of illness?* holds potential – if it is asked with genuine interest rather than as a clinical checkbox; it can open a space for reflection that reaches beyond clinical concerns. Embedded in the question lies recognition; that chronic illness involves not only medical management but also existential negotiation, longing, and the will to persevere. As Murakami (2009) writes; *Pain is inevitable. Suffering is optional*. It is not (only) about running – but about what keeps a person moving. Where healthcare embraces this perspective, it is a place where vulnerability is met with recognition, and where people are supported in finding meaning – not despite their illness, but *with* it as part of life.

*Illness weariness?*

*A steady voice holds unease –*.

*the body remembers rhythm.*

## Data Availability

This article is based on autoethnographic and phenomenologically inspired reflections grounded in the first author’s lived experience with myasthenia gravis. As such, there are no external datasets associated with this manuscript. All insights are derived from the author’s personal and embodied experience and theoretical interpretation. Any further underlying data will be made available upon reasonable request from the corresponding author.
